# Effects of Increased Optic Nerve Sheath Diameter on Inadequate Emergence from Anesthesia in Patients Undergoing Robot-Assisted Laparoscopic Prostatectomy: A Prospective Observational Study

**DOI:** 10.3390/diagnostics11122260

**Published:** 2021-12-02

**Authors:** Yu Jeong Bang, Heejoon Jeong, Burn Young Heo, Byung Seop Shin, Woo Seog Sim, Duk-Kyung Kim, Sang Hyun Lee, Ji Su Kim, Young Hee Shin

**Affiliations:** Samsung Medical Center, Department of Anesthesiology and Pain Medicine, Sungkyunkwan University School of Medicine, 81 Irwon-ro, Gangnam-gu, Seoul 06351, Korea; gagreflex.bang@gmail.com (Y.J.B.); heejoon.jeong@samsung.com (H.J.); burnyoung.heo@samsung.com (B.Y.H.); byungseop.shin@samsung.com (B.S.S.); wooseog.sim@samsung.com (W.S.S.); dk68.kim@samsung.com (D.-K.K.); shsara2t17.lee@samsung.com (S.H.L.); jisu0106@samsung.com (J.S.K.)

**Keywords:** anesthesia recovery period, delayed emergence from anesthesia, emergence delirium, prostatectomy, robotic surgical procedures

## Abstract

(1) Background: Robot-assisted laparoscopic prostatectomy (RALP) is preferred over open prostatectomy because it offers superior surgical outcomes and better postoperative recovery. The steep Trendelenburg position and pneumoperitoneum required in Robot-assisted laparoscopic prostatectomy, however, increase intracranial pressure (ICP). The present study aimed to evaluate the effects of elevated ICP on the quality of emergence from anesthesia. (2) Methods: Sixty-seven patients undergoing RALP were enrolled. We measured optic nerve sheath diameter at four timepoints during surgery. Primary outcome was inadequate emergence in the operating room (OR). Secondary outcomes were postoperative neurologic deficits of dizziness, headache, delirium, cognitive dysfunction, and postoperative nausea and vomiting (PONV). (3) Results: A total of 69 patients were screened for eligibility and 67 patients completed the study and were included in the final analysis. After establishing pneumoperitoneum with the Trendelenburg position, ONSD increased compared to baseline by 11.4%. Of the 67 patients, 36 patients showed an increase of 10% or more in optic nerve sheath diameter (ONSD). Patients with ΔONSD ≥ 10% experienced more inadequate emergence in the OR than those with ΔONSD < 10% (47.2% vs. 12.9%, *p* = 0.003). However, other variables related to the quality of emergence from anesthesia did not different significantly between groups. Similarly, neurologic deficits, and PONV during postoperative day 3 showed no significant differences. (4) Conclusions: ICP elevation detected by ultrasonographic ONSD measurement was associated with a transient, inadequate emergence from anesthesia.

## 1. Introduction

Robot-assisted laparoscopic prostatectomy (RALP) is the preferred alternative to open prostatectomy because it offers superior surgical outcomes and rapid postoperative recovery [1]. To secure the operation field and facilitate surgical access, a steep Trendelenburg position (45°) and pneumoperitoneum with CO_2_ are essential in RALP. However, the Trendelenburg position combined with a pneumoperitoneum adversely influences cardiovascular hemodynamics, respiratory mechanics, and cerebrovascular perfusion during surgery. In particular, significant changes in cerebral hemodynamic physiology leading to increases in intracranial pressure (ICP) and intraocular pressure have been noted [2,3]. A few postoperative complications, such as visual impairment, headache, and confusion have been reported in some case reports [4,5,6,7,8,9].

Optic nerve sheath diameter (ONSD) using ultrasonographic measurement correlated well with directly measured ICP. It is also known as a method of accurately and quickly monitoring changes in ICP [10,11,12,13]. Ultrasonographic optic nerve measurement has emerged as a reliable surrogate for invasive ICP measurement for intraoperative ICP monitoring. ICP changes do not affect ONSD at low levels (ICP 8–10 mmHg), but are distended only when ICP increases obviously [12]. Numerous studies have reported increase in ONSD in patients undergoing laparoscopic gynecologic surgery or RALP [3,14]. Elderly patients lack autoregulation of cerebral blood flow and are unable to tolerate a prolonged elevated ICP [15], so it is necessary to investigate intraoperative ICP elevation and its associated clinical effects in this population. However, data on the effect of elevated ICP as measured by ONSD on clinical outcomes are lacking.

The present study aimed to evaluate the effect of elevated ICP due to position and pneumoperitoneum on quality of emergence from anesthesia. Primary outcome was the occurrence of inadequate emergence according to elevated ICP. Secondary outcomes were other variables associated with emergence quality such as time to eye opening, time to extubation, and inadequate emergence in post-anesthesia care unit (PACU). Additionally, we investigated whether elevated ICP was associated with postoperative nausea or vomiting, and neurologic deficits including dizziness, headache, delirium, and cognitive dysfunction.

## 2. Materials and Methods

This study is a single center prospective observational study. The study was approved by the institutional review board of Samsung Medical Center, Seoul, South Korea (IRB number: SMC 2020-10-132-001, 20 November 2020) and registered at CRIS (https://cris.nih.go.kr, accessed on 20 November 2020; registration number KCT 0005665). 

### 2.1. Study Population and Recruitment

The present study was carried out between December 2020 and March 2021. Included were patients aged over 60 years who were scheduled to undergo robotic pelvic surgery, with American Society of Anesthesiologists physical status Ⅰ–Ⅲ. Exclusion criteria were hemodynamic instability; history of eye surgery; current eyeball disease (e.g., cataract, glaucoma, retinal detachment); increased intracranial pressure (e.g., brain tumor, spinal cord lesion); and current medications known to influence emergence from anesthesia (e.g., benzodiazepines, opioids). We initially planned to enroll either female or male participants undergoing robotic pelvic surgery. However, there were no eligible female participants because surgeons at our hospital overwhelmingly prefer open surgery or simple laparoscopic surgery for elderly female patients. Therefore, only male patients undergoing RALP were enrolled during the study period. A total of 69 patients were screened for eligibility. After obtaining informed consent, we enrolled 67 patients scheduled for elective RALP.

### 2.2. Anesthesia Protocol

During standard monitoring of patients per protocol, general anesthesia was induced with intravenous rocuronium 0.8 mg.kg^−1^ and propofol and remifentanil via target-controlled infusion (Orchestra^®^ Base Primea; Fresenius Kabi, Brezins, France). Attending anesthesiologist used the following ventilator settings: tidal volume of 8 mL·kg^−1^ of ideal body weight, FiO_2_ 0.4, and zero positive end tidal pressure. Respiratory rate was adjusted to maintain an inspiratory peak pressure less than 30 cmH_2_O and an EtCO_2_ between 35 and 38 mmHg. After establishing pneumoperitoneum, ventilator setting was adjusted to a target PaCO_2_ of 32–35 mmHg. Effect site concentrations of propofol and remifentanil were adjusted to achieve bi-spectral index (BIS) values of 40 to 50 and to maintain mean blood pressure and heart rate at within 20% of pre-induction values. Neuromuscular blockade monitoring was performed with an NMT device (Datex-Ohmeda, Madison, WI, USA). Continuous infusion of rocuronium was adjusted to maintain two counts of train of four (TOF) and stopped at least 30 min before the end of surgery. Palonosetron 0.075 mg and propacetamol 1 g were administered for pain control and as antiemetics. When the surgeon started suturing subcutaneous tissue, the propofol infusion was stopped or decreased by targeting a BIS of 60–65. After surgery, residual neuromuscular blockade was reversed with pyridostigmine (250 mcg·kg^−1^) and glycopyrrolate (10 mcg·kg^−1^). Independent anesthetic provider checked the patient’s consciousness and evaluated if the patient met the pre-determined extubation criteria of (1) sufficient reflexes to protect the airway; (2) adequate gas exchange (respiration rate 10–30 breaths per minute and tidal volume ≥ 6 mL/kg); (3) TOF ratio ≥ 0.9; and (4) Observer Assessment of Alertness/Sedation Scale (OAA/S) ≥ 2. Patients were extubated in the operating room (OR) and transferred to a post-anesthesia care unit (PACU).

### 2.3. Ultrasonographic Measurement of Optic Nerve Sheath Diameter

**Figure 1:**
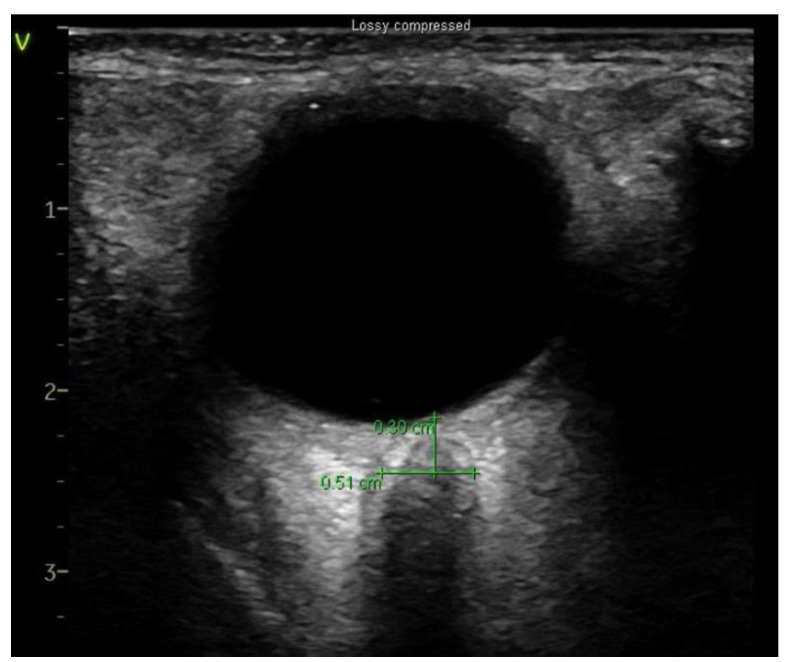
Sonographic optic nerve sheath diameter (ONSD).

A single experienced investigator who has performed > 50 scans measured ONSD using a portable US unit (Sonosite X-Porte, Fujifilm Sonosite, Bothell, WA, USA) with a 5–10 Hz linear probe [16]. When general anesthesia induced and hemodynamically stable conditions were reached, ultra-sonographic measurements were taken. After applying water soluble lubricant, the probe was placed on the closed upper eyelid with minimal pressure in a direction parallel to the eyelid. Three measurements were acquired in the transverse plane of each eye at four timepoints (Figure 1): T0, 10 min after the induction of anesthesia in the supine position; T1, 10 min after introducing pneumoperitoneum (13 mmHg of insufflation pressure) in the steep Trendelenburg position (45° incline); T2, 10 min after releasing the pneumoperitoneum in the steep Trendelenburg position (45° incline); T3, 10 min after position change to a supine position.

Ultrasonographic images were reviewed using a picture archival and communication system (PACS, Centricity Enterprise Web version 3.0; GE Healthcare, Milwaukee, WI, USA) and ONSD was measured 3 mm behind the globe by the blinded two clinicians. After obtaining the mean ONSD of each eye, the mean value of ONSD in both eyes was calculated. We defined ∆ONSD as follows: ∆ONSD = 100 × (maximum ONSD at any time point—ONSD at baseline)/ONSD at baseline.

### 2.4. Data Collection and Outcomes

We recorded physical characteristics, intraoperative variables, and length of stay (LOS) by review of medical records. All patients underwent the Mini Mental State Exam (MMSE) prior to surgery as a baseline examination. The primary outcome was inadequate emergence in OR. The variables related to quality of emergence from general anesthesia, namely time to eye opening, and time to extubation were collected. Inadequate emergence was evaluated using the Richmond Agitation and Sedation Scale (RASS) after extubation by the attending anesthesiologist. Inadequate emergence was classified as agitated emergence or hypoactive emergence. Agitated emergence was defined as a RASS score > 1; hypoactive emergence was defined as a RASS score < −2 [17,18]. Time to eye opening or extubation was defined as the time from the end of surgery to eye opening or extubation. Secondary outcomes included inadequate emergence in the PACU, neurologic deficit, and postoperative nausea and vomiting (PONV) on postoperative day (POD) 3. A team of trained nurses assessed inadequate emergence and delirium in the PACU. Inadequate emergence in the PACU was determined by RASS score 10 min after PACU arrival, and delirium was evaluated using the Nursing Delirium Screening scale (Nu—DESC) [19]. To evaluate neurologic deficits, we collected data on delirium, headache, and dizziness. Postoperative cognitive decline was defined as a decrease in MMSE by 2 points [20]. MMSE was performed in all patients on POD 1. We investigated the quality of post-operative recovery using the Quality of Recovery-15 (QoR-15) questionnaire on POD 3 [21].

### 2.5. Sample Size Calculation and Statistical Analysis

Sample size was calculated based on a previous study. Inadequate emergence incidence reported in the literature is 8.2% [18]. The mean value (Standard deviation) of ONSD derived from the existing literature is 4.9 mm (0.4 mm) [22]. We assumed the difference of about 10% was clinically significant considering standard deviations of the results of previous studies. Based on this, we hypothesized that the ONSD value would have to increase by 10% compared to baseline to detect clinically meaningful differences, and inappropriate emergence would increase by 30% in patients with increased ONSD [22]. We calculated that a minimal sample size of 60 was required to achieve a power of 0.8 and α value of 0.05. Our target was to enroll 67 patients to account for an expected 10% attrition rate.

Data are presented as means (standard deviations (SD)) or as medians (inter-quartile ranges (IQR)), as appropriate. The Shapiro–Wilk test was used to explore normality. Significance of differences between groups was assessed using the chi-square test or Fisher’s exact test for categorical variables and T tests or Wilcoxon’s rank sum test for continuous variables as appropriate. Post hoc analyses were also performed. Statistical significance was defined as a *p*-value < 0.05. All analyses were performed using SAS version 9.4 (SAS Institute Inc., Cary, NC, USA).

## 3. Results

**Figure 2:**
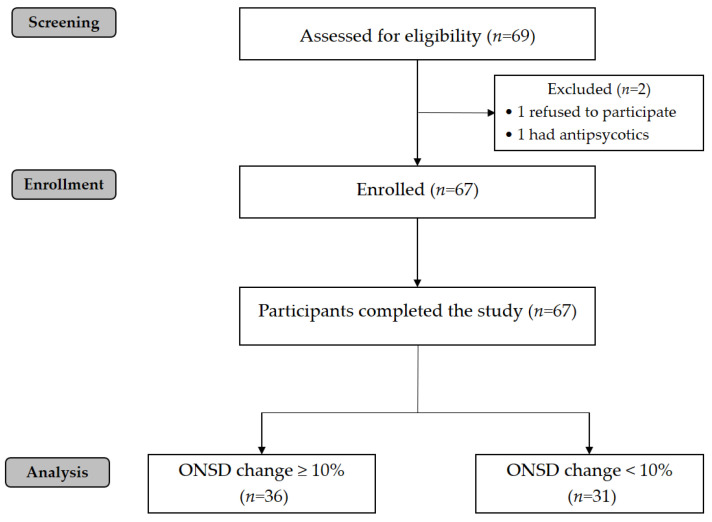
STROBE flow chart of participants. STROBE, Strengthening the Reporting of Observational Studies in Epidemiology formatting.

**Figure 3:**
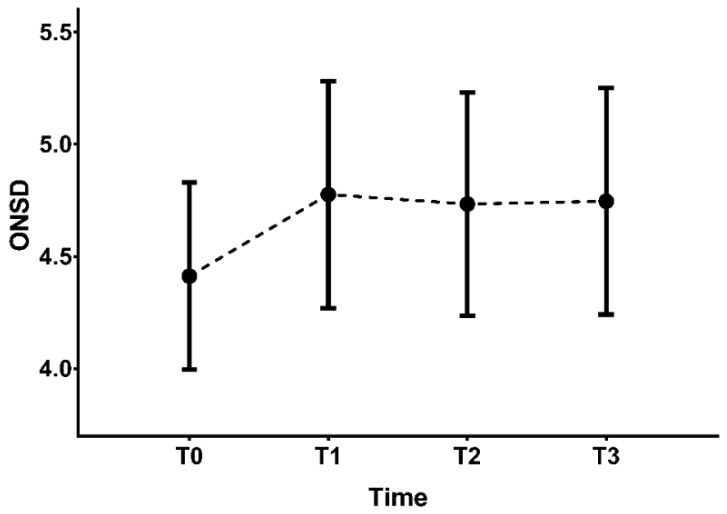
Changes in ONSD during robot-assisted laparoscopic prostatectomy. T0, 10 min after anesthetic induction in the supine position; T1, 10 min after establishing pneumoperitoneum with steep Trendelenburg position; T2, 10 min after de-sufflation of pneumoperitoneum; T3, end of surgery in the supine position.

A total of 69 patients were screened for eligibility. After excluding two patients, 67 patients completed the study and were analyzed (Figure 2). Patients’ demographic and pre-operative data are shown in Table 1. Of the 67 patients, 36 patients showed an increase of 10% or more in ONSD. Thirty-one patients had an increase of less than 10% or no change in ONSD. The trend in ONSD and intraoperative data are shown in Figure 3. At baseline (T0), ONSD was not significantly different between groups. After establishing pneumoperitoneum with the patient in the Trendelenburg position, optic nerve sheath diameter began to increase compared to T0. By the end of surgery, ONSD had not decreased to baseline levels in patients with an ONSD increase of 10% or more. Intra operative variables including BIS, other hemodynamic variables at each time points are not different significantly between groups. (Table 2.)

**Figure 4:**
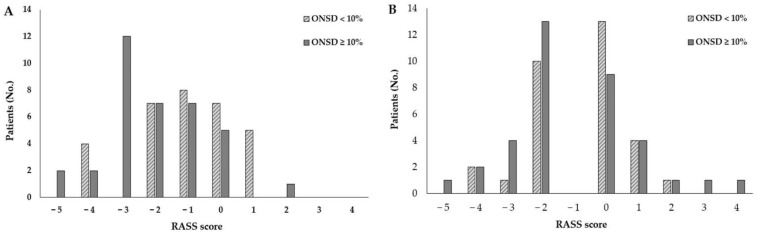
RASS score in OR and upon PACU arrival. (**A**) RASS score in OR; (**B**) RASS score upon PACU arrival. RASS, Richmond Agitation-Sedation Scale.

Patients with ΔONSD ≥ 10% had more inadequate emergence in OR than those with ΔONSD < 10% (47.2% vs. 12.9%, respectively, *p* = 0.003). Figure 4 demonstrates RASS score distribution at two timepoints; after extubation in OR (Figure 4a), and PACU arrival (Figure 4b). Of the two types of inadequate emergence, only hypoactive emergence in patients with ΔONSD ≥ 10% had a significantly higher frequency than in patients with ΔONSD < 10% (44.4% vs. 12.9%, *p* = 0.005). Other variables related to the quality of emergence did not different significantly between groups (Table 3). There was no difference in time to RASS 0 and inadequate emergence in the PACU. Similarly, POCD, neurologic deficit, and PONV during POD 3 were not significantly different between ΔONSD ≥ 10% and ΔONSD < 10% groups. There were no differences in QoR-15 or LOS between groups. 

## 4. Discussion

In this study, patients who underwent RALP had an 11.4% increase in ONSD during pneumoperitoneum in the steep Trendelenburg position. Patients with ΔONSD ≥ 10% had inadequate emergence as assessed using RASS in the OR. However, this unfavorable association soon disappeared and did not seem to affect the quality of emergence upon PACU arrival, PONV, or other neurologic deficits in the PACU or ward on POD 3.

Similar to the results of a previous study, our results confirmed that ONSD increases in the Trendelenburg position with pneumoperitoneum [5,23,24]. The increase in ONSD during surgery means sustained elevation of ICP in the corresponding period. The threshold of ONSD corresponding to IICP (ICP > 20 mmHg) in previous studies varies from 5.22 to 5.8 mm [22,25,26]. The various cut-off values of ONSD are likely to be due to differences in brain injury, race, age, height, and ONSD measurement techniques. However, it was difficult to refer to the cutoff value of neurosurgical patients to evaluate anesthetized patients without intracranial pathology. Therefore, we grouped the patients using ONSD change ratio (10% or more vs. less than 10%) rather than the unclear cut off value and compared the primary outcome between groups. It is a sufficient criterion to determine an increase in ONSD (ICP elevation) considering intra-observer variation (0.2 mm) [12]. Of the 67 patients included in the final analysis, 36 patients had an ONSD increase of 10% or more and only five patients showed increased values of ONSD (>5.7 mm), corresponding to ICP > 20 mmHg during surgery [27]. We conducted further analysis to compare postoperative outcomes in the group with intraoperative ONSD ≥ 5.7 m to those in the group with ONSD < 5.7 mm. There were no significant differences in the variables including the quality of emergence from general anesthesia, neurologic deficits, and PONV.

There were no differences in time to extubation or time to eye opening when patients with ΔONSD ≥ 10% and ΔONSD < 10% were compared; significant differences in inadequate emergence were observed only after extubation. These results suggest that the increase in ONSD can potentially predict inadequate emergence, excluding the effects of neuromuscular blockade. The difference between groups in inadequate emergence disappeared 10 min after PACU arrival, which suggests that the adverse effects of elevated ICP, identified by an increased ONSD of 10% or more, persist only for a short period.

For the result of PONV and postoperative headache, our study showed that there are no association with ONSD. This finding contrasts with Yilmaz et al., who reported the correlation of extent of the increase in ONSD and PONV or postoperative headache in patients undergoing laparoscopic hysterectomy [5]. This discrepancy is probably due to the fact that our study enrolled only elderly male patients, excluding female patients vulnerable to PONV. 

Inadequate emergence after surgery is a common complication in elderly patients [18,28]. Inadequate emergence is associated with postoperative complications and an unpleasant patient experience both of which can impede post-operative recovery and increase medical costs. Although the mechanism of inadequate emergence is unclear, the results of the present study suggest that it could be associated with ICP control. Furthermore, elderly patients are also known to be susceptible to elevated ICP [15], so ICP monitoring with ocular sonography could be helpful for diagnostic and therapeutic purposes as a point of care test during anesthesia. If ICP elevation is suspected based on ONSD measurement, the anesthesiologist could alter management to control the MAP, ventilator settings, CO_2_ de-sufflation or reduction in the angle of the steep Trendelenburg position.

Our study was initiated due to concerns that an elevated ICP during surgery may affect emergence from anesthesia or postoperative neurologic outcomes. However, we found no differences in neurologic outcomes, only inadequate emergence, according to increased ONSD. Fortunately, the inadequate emergence did not last for a long time, so ICP elevation during surgery does not appear to have a permanent effect on psychological or cognitive function. This may be because, in the present study, participants showed an ONSD increase of 11.4% during RALP, which is lower than the values of around 17% reported in previous studies [14,23]. In the present study, EtCo2 and MAP were strictly controlled to achieve target range, and total IV anesthetics was used instead of volatile anesthetics [29]. This could have minimized the increase in ONSD and elevation of ICP, while simultaneously minimizing the effects of increased ONSD on neurologic deficits and emergence from anesthesia. In addition, we enrolled patients without intracranial pathology. Elevated ICP might not result in cerebral parenchymal edema in this population who are assumed to have an intact blood brain barrier. 

In this study, data were collected prospectively and anesthesia and postoperative care were conducted according to standardized protocols. Anesthesia, measurement of intraoperative ONSD, and postoperative outcomes collection were performed by independent blinded anesthesiologists or anesthetic team nurses, respectively. Compared to previous studies that have focused mainly on the pattern of increasing ONSD in the same population, we further evaluated the effects of elevated ONSD on emergence from anesthesia and neurologic outcomes. As medical care advances and population aging progresses, robot assisted surgery for the elderly has become more popular. The elderly patients lack the autoregulation of cerebral blood flow, and are unable to tolerate prolonged elevated ICP [15]. Therefore, it is necessary to further investigate the correlation between the clinical outcome and the inevitable elevated ICP caused by surgical position and pneumoperitoneum. To the best of our knowledge, this is the first comprehensive study to prospectively investigate the effects of the elevated ICP as evaluated by ONSD on neurologic outcomes and emergence after surgery.

Despite these strengths, this study had several limitations. First, only male elderly patients with a normal ICP were included in the study. Results can therefore not be generalized to pediatrics, female patients, or patients at risk of elevated ICP. Second, although sample size was derived by referring to previous studies, calculated sample size might be relatively small. For sample size calculation, we arbitrarily assumed 10% of increase in ONSD as a dividing point. Because there was no clear reference in a similar setting, we had no choice but to make a clinical assumption. In the further investigation, including the predictability of ONSD for inadequate emergence or neurological complications, it would be a better plan to include a large number of participants. Third, tracheal extubation commenced immediately after extubation criteria were met. Although, tracheal extubation was performed after consciousness and neuromuscular block were sufficiently confirmed according to pre-determined criteria, patients had become drowsy (RASS < −3) immediately before discharge from OR. We considered that this might be a unique characteristic of the elderly that occurs in the process of emergence from general anesthesia. However, it is necessary to introduce more stringent extubation criteria to perfectly exclude residual effects of anesthetics. Last, neurological outcomes were collected up to POD 3 so we were not able to determine the association between an increase in ONSD during surgery and long-term neurologic deficits after surgery. We evaluated postoperative cognitive impairment using only MMSE, but a more detailed neurocognitive battery is required for accurate diagnosis.

The present study showed a potential link between elevated ICP and inadequate emergence. To assess other effects of elevated ICP on neurological outcomes, further research is needed, including more detailed neurologic tests and long term follow-up.

## 5. Conclusions

The patients undergoing RALP showed an increase in ONSD during surgery. ICP elevation detected by ultrasonographic ONSD measurement was associated with inadequate emergence from anesthesia in the OR. We suggest that ICP monitoring using sonographic ONSD measurement is helpful to identify patients who may be at increased risk of inadequate emergence or morbidity due to increased ICP. 

## Figures and Tables

**Table 1:** Patient characteristics and perioperative data.

Parameter	All Patient (*n* = 67)	ΔONSD < 10% (*n* = 31)	ΔONSD ≥ 10% (*n* = 36)	*p*-Value
Age (years)	68.0 ± 4.8	68.4 ± 5.0	67.8 ± 4.6	0.590
Body mass index (kg/m^2^)	25.1 ± 3.0	25.0 ± 3.6	25.2 ± 2.4	0.789
ASA physical status (I/II/Ⅲ)	10/43/14	5/19/7	5 / 24 /7	0.900
Hypertension	41/67 (61.2%)	19/31 (61.3%)	22/36 (61.1%)	0.988
Diabetes mellitus	9/67 (13.4%)	4/31 (12.9%)	5/36 (13.9%)	>0.999
Dyslipidemia	18/67 (26.9%)	9/31 (29.0%)	9/36 (25.0%)	0.710
Renal dysfunction	2/67 (3.0%)	1/31 (3.2%)	1/36 (2.8%)	>0.999
Hepatic dysfunction	2/67 (0.3%)	1/31 (3.23%)	1/36 (2.8%)	>0.999
PreOP hemoglobin	13.4 (12.8, 14)	13.4 (13, 14)	13.5 (12.65, 14.05)	0.910
MMSE pre	28 (27, 29)	28 (27, 29)	28 (27, 29)	0.703
Anesthetic time (min)	203.0 ± 46.5	207.5 ± 57.2	199.2 ± 35.2	0.487
Pneumoperitoneum time (min)	140 (111, 168)	140 (108, 171)	142 (115.5, 162)	0.945
Crystalloid (mL)	750 (700, 1000)	700 (650, 1000)	800 (700, 950)	0.463
EBL (mL)	200 (100, 250)	150 (100, 250)	200 (150, 250)	0.138
Propofol (mg)	1500 (1240, 1800)	1540 (1260, 1840)	1400 (1240, 1750)	0.673
Remifentanil (mg)	0.9 (0.75, 1.1)	0.95 (0.75, 1.15)	0.9 (0.75, 1.08)	0.583
Rocuronium (mg)	100 (91, 114)	100 (95, 120)	102.85 (90, 110)	0.326
Opioid-PACU *	4.67 (3.33, 6.5)	4 (3.33, 5.33)	4.83 (3.67, 6.83)	0.336

Values are expressed as mean ± SD, mean (IQR), or as number of patients. * Opioid consumption was converted to morphine equivalent dose. Abbreviations: ASA, American Society of Anesthesiologists; EBL, estimated blood loss.

**Table 2:** Intraoperative variables.

Parameters	All Patients (*n* = 67)	ΔONSD < 10% (*n* = 31)	ΔONSD ≥ 10% (*n* = 36)	*p*-Value
BIS				
T0	43 (34, 50)	41 (33, 48)	45 (38.5, 50.5)	0.256
T1	39 (33, 43)	37 (31, 40)	40 (35, 44.5)	0.048 *
T2	45 (40, 53)	47 (40, 56)	43.5 (40, 50)	0.281
T3	59 (52, 65)	59 (49, 67)	59 (52, 64.5)	0.940
Heart rate				
T0	74 (62, 85)	78 (67, 86)	70 (60.5, 84.5)	0.176
T1	62 (56, 69)	63 (57, 69)	62 (56, 71)	0.584
T2	59 (55, 67)	59 (54, 64)	59.5 (55, 69.5)	0.567
T3	62 (55, 72)	62 (55, 76)	61.5 (55, 71.5)	0.443
Mean blood pressure				
T0	79 (70.33, 88.33)	78 (64.67, 87)	79 (71.67, 89.83)	0.980
T1	87 (79, 98)	87 (79, 98.33)	89 (79.33, 95.33)	0.692
T2	83.33 (74.33, 92.33)	83 (72.33, 92.33)	83.33 (74.5, 92.17)	0.871
T3	89.33 (78, 98.33)	91.67 (78, 102)	88.33 (78.5, 96)	0.389
Peak inspiratory pressure				
T0	15 (13, 16)	15 (13, 18)	14 (12.5, 16)	0.160
T1	23 (20, 25)	24 (21, 26)	23 (19.5, 25)	0.252
T2	16 (14, 19)	17 (14, 20)	15.5 (13, 18)	0.214
T3	16 (14, 18)	16 (14, 18)	15 (13, 18)	0.380
Core body temperature				
T0	36 (35.8, 36.2)	36.1 (35.9, 36.3)	36 (35.75, 36.2)	0.119
T1	36 (35.8, 36.3)	36 (35.8, 36.3)	36 (35.75, 36.2)	0.337
T2	36 (35.6, 36.1)	35.9 (35.5, 36.1)	36 (35.6, 36.1)	0.311
T3	36 (35.6, 36.1)	35.9 (35.5, 36.1)	36 (35.6, 36.1)	0.426
End tidal CO_2_				
T0	34 (33, 36)	35 (33, 36)	33.5 (32.5, 35.5)	0.259
T1	35 (34, 36)	35 (34, 36)	35 (34, 35)	0.322
T2	36 (35, 37)	36 (34, 37)	36 (35.5, 38)	0.149
T3	36 (35, 38)	37 (35, 38)	36 (34, 38)	0.205
Optic nerve sheath diameter (ONSD)				
T0	4.4 (4.15, 4.7)	4.45 (4.15, 4.75)	4.35 (4.05, 4.6)	0.190
T1	4.8 (4.4, 5.1)	4.55 (4.2, 4.95)	4.88 (4.58, 5.13)	0.110
T2	4.7 (4.3, 5.05)	4.65 ( 4.25, 4.95)	4.78 (4.45, 5.1)	0.182
T3	4.75 (4.3, 5.1)	4.6 (4.15, 4.95)	4.88 (4.45, 5.18)	0.034 ^*^
Δ ONSD (%)				
Δ ONSD T0–T1	7 (3, 13)	4 (1, 7)	12 (9, 16)	<0.001 *
Δ ONSD T0–T2	−1 (−3, 2)	0 (−3, 2)	−1 (−5, 3)	0.567
Δ ONSD T0–T3	5 (1, 14)	2 (0, 5)	14 (3, 19)	<0.001 *
Δ ONSD max	10 (5, 16)	5 (2, 7)	16 (12, 21)	<0.001 *

Values are expressed as mean ± SD or median (IQR). T0, 10 min after anesthetic induction in the supine position; T1, 10 min after establishing pneumoperitoneum in the steep Trendelenburg position; T2, 10 min after de-sufflation of the pneumoperitoneum; T3, at the end of surgery in the supine position. * *p*-value < 0.05.

**Table 3:** Composite variables to assess the quality of emergence and neurologic deficits.

Parameters	All Patients (*n* = 67)	ΔONSD < 10% (*n* = 31)	ΔONSD ≥ 10% (*n* = 36)	*p*-Value
Operating room				
Inadequate emergence	21 (31.3%)	4 (12.9%)	17 (47.2%)	0.003 *
Hypoactive emergence	20 (29.9%)	4 (12.9%)	16 (44.4%)	0.005 *
Agitated emergence	1 (1.5%)	0 (0%)	1 (2.8%)	>0.999
Time to eye opening (min)	9 (5, 12)	7 (4, 12)	9 (5.5, 12)	0.615
Time to extubation (min)	9 (6, 11)	9 (6, 12)	9.5 (7, 11)	0.557
PACU				
Inadequate emergence	14 (20.9%)	4 (12.9%)	10 (27.8%)	0.228
Hypoactive emergence	10 (14.9%)	3 (9.7%)	7 (19.4%)	0.320
Agitated emergence	4 (6.0%)	1 (3.2%)	3 (8.3%)	0.618
Time to RASS 0 (min)	35 (27, 47)	34 (22, 47)	37 (30, 46.5)	0.379
Neurologic deficits	9 (13.4%)	3 (9.7%)	6 (16.8%)	0.488
Delirium	4 (6.0%)	1 (3.2%)	3 (8.3%)	0.618
Headache	2 (3.0%)	0 (0%)	2 (5.6%)	0.495
Dizziness	3 (4.5%)	2 (6.5%)	1 (2.8%)	0.592
PONV	2 (3.0%)	2 (6.5%)	0 (0%)	0.210
PACU stay (min)	63 (57, 69)	62 (58, 67)	64 (56, 72)	0.521
Ward				
Neurologic deficits	18 (26.9%)	10 (32.3%)	8 (22.2%)	0.356
POCD	13 (19.4%)	8 (25.8%)	5 (14.0%)	0.219
Delirium	4 (6.0%)	2 (6.5%)	2 (5.6%)	>0.999
Headache	4 (6.0%)	3 (9.68%)	1 (2.8%)	0.329
Dizziness	14 (20.9%)	7 (19.4%)	7 (22.6%)	0.753
PONV	18 (26.9)	9 (29.0%)	9 (25.0%)	0.710
Recue analgesics (No.)	2/3/1	1//2/1	1/1/0	0.697
QoR -15	129 (111, 134)	130.5 (111, 136)	129 (112, 132.5)	0.634
LOS (day)	7 (6, 8)	7 (6, 8)	7 (6, 8)	0.249

Values are expressed as mean ± SD, median (IQR), or as number of patients (%). PACU, post-anesthesia care unit; PONV, postoperative nausea and vomiting; POCD, postoperative cognitive dysfunction; QoR-15, Quality of Recovery 15; LOS, length of stay. * *p*-value < 0.05.
